# Disparities in Outcomes of Hospitalizations Due to Multiple Myeloma: A Nationwide Comparison

**DOI:** 10.7759/cureus.47319

**Published:** 2023-10-19

**Authors:** Sushmita Khadka, Swetha Balaji, Japjeet Kaur, Dhanshree Solanki, Mariia Kasianchyk, Humayra Chowdhury, Ishani Patel, Muhammad Qasim, Maheshkumar Desai, Prakash Maiyani, Dharmeshkumar V Moradiya, Darshan Lal, Achint A Patel, Manidhar Lekkala

**Affiliations:** 1 Internal Medicine, Guthrie Robert Packer Hospital, Sayre, USA; 2 Medicine, Scripps Clinic John R. Anderson V Medical Pavilion, San Diego, USA; 3 Medicine, Sub-divisional Civil Hospital, Ajnala, IND; 4 Hospital Administration, Rutgers University, New Brunswick, USA; 5 Medicine, Vinnytsia National Medical University, Vinnytsia, UKR; 6 Medicine, Mount Sinai Health System, New York, USA; 7 Medicine, Houston Medical Center, Warner Robins, USA; 8 Internal Medicine, Hospital Corporation of America (HCA) Healthcare/University of South Florida (USF) Morsani College of Medicine Graduate Medical Education (GME) Oak Hill Hospital, Brooksville, USA; 9 Internal Medicine, Hamilton Medical Center, Medical College of Georgia/Augusta University, Dalton, USA; 10 Internal Medicine, Gold Coast University Hospital, Southport, AUS; 11 Internal Medicine, St. John of God Murdoch Hospital, Murdoch, AUS; 12 Hospital Medicine, Geisinger Commonwealth School of Medicine, Scranton, USA; 13 Oncology, University of Kansas, Kansas City, USA

**Keywords:** multiple myeloma, length of stay, newest treatment for multiple myeloma, multiple myeloma prognosis, multiple myeloma treatment, temporal trends

## Abstract

Background

With the advent of novel treatments, there is a declining trend in the multiple myeloma (MM) mortality rate with an increasing hospitalization rate. However, there is limited population-based data on trends and outcomes of hospitalizations due to MM in the United States (US).

Methods

We analyzed the publicly available Nationwide Inpatient Sample (NIS) from 2007 to 2017 to identify MM hospitalizations.

Results

Hospitalizations for MM increased from 17,100 (8.71%) in 2007 to 19,490 (9.92%) in 2017. The in-hospital mortality rate declined from 8.4% in 2007 to 4.9% in 2017 (P <0.001) and discharge to facilities decreased from 20.4% in 2007 to 17.4% in 2017 (P <0.001). The odds of in-hospital mortality were higher with increasing age (odds ratio (OR): 1.46; 95% confidence interval (CI): 1.38 -1.54; P <0.0001), pneumonia (OR: 4.18; 95% CI: 3.63 - 4.81, P <0.0001), septicemia (OR: 2.50; 95% CI: 2.22 - 2.82; P <0.0001), renal failure (OR: 1.48; 95% CI: 1.34 -1.64; P <0.0001), uninsured/self-pay insurance status (OR: 2.69; 95% CI: 2.18 - 3.3; P <0.0001), rural hospital (OR: 2.26; 95% CI: 1.88 -2.72; P<0.0001), and urban-non-teaching hospitals (OR: 1.38; 95% CI: 1.23 - 1.56; P <0.0001). Also, increasing age (OR: 1.14; 95% CI: 1.11-1.18, P <0.0001), Black race (OR: 1.12; 95% CI: 1.02-1.23, P <0.0001), and multiple comorbidities were associated with higher disability.

Conclusion

Hospitalizations for MM continued to increase, whereas in-hospital mortality continued to decrease. Advanced age, sepsis, pneumonia, and renal failure were associated with higher odds of mortality in MM patients.

## Introduction

Multiple myeloma (MM) is typically characterized by the neoplastic proliferation of plasma cells producing a monoclonal immunoglobulin. The plasma cells proliferate in the bone marrow and result in extensive skeletal destruction with osteolytic lesions, osteopenia, and/or pathologic fractures. It is the second-most common hematologic malignancy. Yearly, almost half a million people are affected worldwide. Multiple myeloma is more prevalent in males, in patients older than 60 years of age, and has a median survival of six to seven months without treatment [1]. In 2017, 140,779 people lived with MM. It is more common in men, the Black population, and individuals with monoclonal gammopathy of undetermined significance (MGUS) [2]. The prevalence of MM in the US increased from 50,484 cases in 2002 to 118,539 cases in 2014, per the Surveillance, Epidemiology, and End Results (SEER) database [3]. There are many factors that influence hospitalization rates, starting with complications of the disease, co-morbid conditions, hospital type, insurance, and other factors [1]. Older patients with MM present more often with advanced International Staging System (ISS) stages and have significantly shorter survival times than younger patients [4].

The rate of adverse hospital discharge remained similar, at 20% in 2007 compared with 18% in 2017 (P = 0.08) [5]. From 2010 to 2016, the five-year relative survival for multiple myeloma was 53.9%. To put that statistic into perspective, the five-year relative survival rate for multiple myeloma in 1998, the year the Multiple Myeloma Research Foundation (MMRF) was founded, was just 34.6% [6]. Patient outcomes have shown significant improvement in the last two decades, after the introduction of proteasome inhibitors and immunomodulatory drug therapies. Yet, there are patients who have poor outcomes who contribute around 20%-30% of all cases [7,8]. Despite the treatment options available, there is a gap between the efficacy of treatment in a clinical trial setting and the results it yields in real-world practice [9]. Outcomes have shown a dramatic improvement with novel treatments, with progression-free survival rates of approximately 24 to 30 months in patients not receiving maintenance therapy. Still, there is a subset of patients who do not respond to the therapy, which might be affecting the outcomes. In order to improve the outcomes of patients who are at high risk, it is crucial to identify them at the time of presentation [8]. There are studies on the incidence and prevalence of MM; however, the association of hospitalization trends with different variables and the outcomes of such hospitalizations is yet to be studied in detail [10]. The focus of this study is to determine hospitalization rates, factors affecting hospitalizations, and reasons contributing to poor outcomes of the disease.

## Materials and methods

Data source

We extracted our study cohort from the Nationwide Inpatient Sample (NIS) database of the Healthcare Cost and Utilization Project (HCUP), Agency for Healthcare Research and Quality (AHRQ) [11]. The NIS is the largest all-payer publicly available database on inpatient discharges from US hospitals, maintained by the AHRQ [11]. The NIS approximates a 20% stratified sample of discharges from US community hospitals, excluding rehabilitation and long-term acute care hospitals, and contains more than seven million hospitalizations annually [11]. With the established weights in NIS, these data could be weighted to represent the standardized US population and obtain national estimates with high accuracy [12].

Study population and design

We queried the 2007-2017 NIS database using the International Classification of Diseases, 9th Revision, Clinical Modification, and International Classification of Diseases, 10th Revision, Clinical Modification (ICD-9/10-CM) diagnoses codes for MM. These codes have been used by previously published articles from administrative databases such as NIS. We extracted demographics, hospital-level characteristics (geographical region, size, and teaching status), and patient-level characteristics as supplied as part of NIS [13]. We estimated comorbidities using Elixhauser comorbidity software (Refined for ICD-10-CM Healthcare Cost and Utilization Project (HCUP), December 2022, Agency for Healthcare Research and Quality, Rockville, MD) and mortality risk using the validated All Patient Refined Diagnosis Related Groups (APR-DRGs) severity score, which is also supplied by HCUP tools and software [14,15]. Specific concurrent medical conditions and procedures of interest were identified by ICD-9/10-CM diagnosis and procedure codes.

Statistical analysis

To establish the trend, we calculated the proportion of hospitalizations due to the primary diagnosis of MM. Descriptive statistics were performed to present the baseline difference in sociodemographic, comorbidity, and hospital-level characteristics. Outcomes of interest were discharge, disposition of the hospitalizations, and length of stay (LOS). We studied the temporal trends of the outcomes of hospitalizations due to MM over the study period, and the Cochrane Armitage Trend test was used to analyze the trend. We compared categorical variables with the chi-square test and continuous variables were compared with the Student's t-test or Wilcoxon rank-sum test. To estimate the predictors of poor outcomes (in-hospital mortality and APR-DRG-defined disability), we used logistic regression models and adjusted them for potential confounders. We utilized SAS version 9.3 (SAS Institute, Cary, NC) for all analyses and included designated weight values to produce nationally representative estimates [12]. For regression models, we used survey procedures to account for the inherent survey design of NIS to produce more robust estimates [16]. We considered a two-tailed p-value <0.05 as statistically significant.

## Results

**Figure 1:**
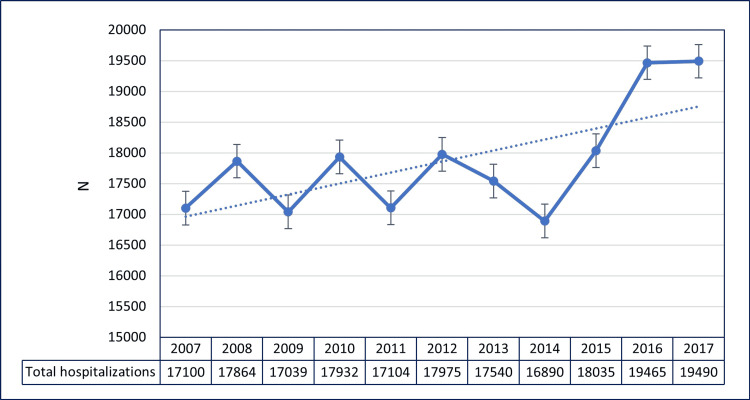
Temporal trends of hospitalizations due to multiple myeloma

A total of 196,433 hospitalizations due to MM from 2007 to 2017 were identified. There was an increase in hospitalizations due to MM from 17,100 (8.7%) in 2007 to 19,490 (9.9%) in 2017 (Figure 1).

Baseline characteristics of the study cohort

The median age of hospitalization was 64 years, with an interquartile range (IQR) of 56-72. The highest percentage of patients hospitalized were in the age group of 50-64 years (39.3%). Males had a higher hospitalization rate than females (55.4% vs. 44.5%). The hospitalization rate was highest among Caucasian patients (56.5%), followed by Black patients (20.0%). Among the hospitalized patients, a considerable number were hypertensive (56.5%). Other significant comorbidities included fluid and electrolyte disorders (44.7%), anemia deficiency (39.4%), and renal failure (25.8%). There was a prominent difference in hospitalization rates between urban/non-teaching hospitals and rural hospitals (21.4% vs. 5.0%); 34.9% of patients were admitted electively when compared to 65.1% of patients who required emergency admission. Among the hospital regions, the South had the highest percentage of hospitalizations (36.6%). Admissions were higher on weekdays as compared to weekends (85.4% and 14.6%, respectively). Large-bed hospitals had the highest percentage of admissions (69.1%). A detailed description of baseline characteristics can be found in Table 1.

**Table 1:** Baseline characteristics of hospitalization due to multiple myeloma †This represents a quartile classification of the estimated median household income of residents in the patient's zip code. These values are derived from zip code demographic data obtained from Claritas. The quartiles are identified by values of one to four, indicating the poorest to wealthiest populations. Because these estimates are updated annually, the value ranges vary by year. HMO: health maintenance organization

Characteristics	N	Percentage
Overall	196,433	100
Age, in years (mean ± SE)		65 (0.2)
Age, in years (median [q1-q3])		64 (56-72)
Age, in years (%)		
18-34	1,018	0.5
35-49	17,540	8.9
50-64	77,196	39.3
65-79	76,512	39
>=80	24,168	12.3
Gender (%)		
Male	108,897	55.4
Female	87,407	44.5
Race (%)		
White	111,044	56.5
Black	39,272	20
Hispanic	15,729	8
Others	10,887	5.5
Missing	19,483	9.9
Comorbidities (%)		
Obesity	13,850	7.1
Hypertension	111,010	56.5
Diabetes mellitus with chronic complications	9,864	5
Diabetes mellitus without chronic complications	30,231	15.4
Congestive heart failure	19,379	9.9
Valvular heart disease	7,349	3.7
History of chronic pulmonary disease	22,077	11.2
Pulmonary circulatory disease	4,631	2.4
Peripheral vascular disease	5,250	2.7
Paralysis	4,857	2.5
Coagulopathy	36,014	18.3
Solid tumor without metastasis	3,032	1.5
Lymphoma	1,798	0.9
Metastatic cancer	5,941	3
Weight loss	22,762	11.6
Liver disease	4,244	2.2
Alcoholism	2,050	1
Other neurological disorders	11,567	5.9
Renal failure	50,702	25.8
Hypothyroidism	20,089	10.2
Arthritis	3,145	1.6
Anemia deficiency	77,430	39.4
Fluid and electrolyte disorders	87,711	44.7
Depression	19,377	9.9
Psychoses	5,581	2.8
Drug abuse	2,446	1.3
AIDS	326	0.2
Peptic ulcer disease	430	0.2
Median household income† (%)		
1st quartile	50,638	25.8
2nd quartile	46,584	23.7
3rd quartile	47,538	24.2
4th quartile	47,556	24.2
Primary insurance (%)		
Medicare/Medicaid	113,818	57.9
Private including HMO	70,475	35.9
Uninsured/Self-pay	11,613	5.9
Hospital bed size (%)		
Small	21,591	11
Medium	38,403	19.6
Large	135,678	69.1
Hospital type (%)		
Rural	9,743	5
Urban/Non-teaching	42,043	21.4
Teaching	143,885	73.3
Hospital region (%)		
Northeast	43,501	22.2
Midwest	47,322	24.1
South	71,872	36.6
West	33,738	17.2
Day of admission		
Weekday	167,829	85.4
Weekend	28,604	14.6
Source of admission (%)		
Transfer from another hospital or other health facility	113,656	57.9
Emergency department	82,778	42.1
Type of admission (%)		
Emergent or urgent	127,450	65.1
Elective	68,191	34.9

Outcomes

**Figure 2:**
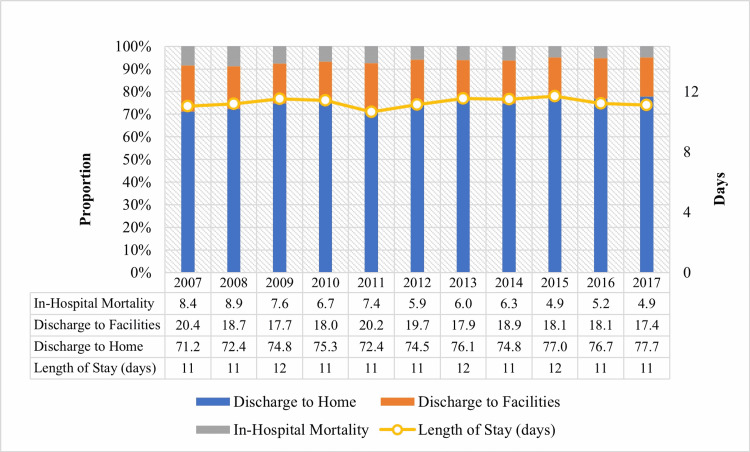
Temporal trends in discharge disposition of hospitalizations due to multiple myeloma

We categorized the discharge disposition of hospitalizations into three categories: discharge to home, discharge to facility, and in-hospital mortality. Out of the total hospitalizations from 2007 to 2017, 74.8% were discharged to home, 18.6% were discharged to a facility, and 6.5% died during the hospitalization. In-hospital mortality declined from 8.4% in 2007 to 4.9% in 2017 (P <0.001), and the discharge-to-facility rate decreased from 20.3% in 2007 to 17.3% in 2017 (P <0.001). The mean LOS was 12 days during the study period (Figure 2).

Predictors of in-hospital mortality

In multivariable regression analysis, increasing age (odd’s ratio (OR): 1.45; 95% CI: 1.3-1.5; p-value <0.0001), conditions such as pneumonia (OR: 4.18; 95% CI: 3.63- 4.81, P <0.0001), septicemia (OR: 2.50; 95% CI: 2.22- 2.82; P <0.0001), and renal failure (OR: 1.48; 95% CI: 1.34-1.64; P <0.0001), were associated with higher in-hospital mortality. Additionally, with respect to insurance status and hospital type, uninsured/self-pay insurance status (OR: 2.69; 95% CI: 2.18-3.3; P <0.0001), rural hospitals (OR: 2.26; 95% CI: 1.88-2.72; P<0.0001), and urban-non-teaching hospitals (OR: 1.38; 95% CI: 1.23-1.56; P <0.0001) had higher odds of in-hospital mortality. A detailed description of predictors is given in Table 2.

**Table 2:** Predictors of in-hospital mortality among multiple myeloma patients HMO: health maintenance organization

Independent variable/characteristic	Odds ratio (95% CI)			P-value
Age (10-year increase)	1.46 (1.38 - 1.54)			<0.0001
Gender				
Male	0.94 (0.85 - 1.03)			0.16
Female	Referent			
Race				
White	Referent			
Black	1.05	(0.94 - 1.18)		0.38
Hispanic	1.05	(0.88 - 1.26)		0.59
Others	1.11	(0.91 - 1.35)		0.31
Comorbidities				
Obesity	0.62 (0.50 - 0.77)			<0.0001
Hypertension	0.59 (0.53 - 0.65)			<0.0001
Diabetes mellitus	1.03 (0.91 - 1.17)			0.65
Congestive heart failure	1.65 (1.44 - 1.88)			<0.0001
Hospital-onset pneumonia	4.18 (3.63 - 4.81)			<0.0001
Septicemia	2.50 (2.22 – 2.82)			<0.0001
History of chronic pulmonary disease	0.98 (0.85 - 1.12)			0.74
Pulmonary circulatory disease	1.43 (1.12 - 1.84)			0.004
Peripheral vascular disease	1.08 (0.84 - 1.38)			0.57
Neurological disease	1.31 (1.11 - 1.56)			0.002
Paralysis	1.86 (1.45 - 2.37)			<0.0001
Coagulopathy	1.61 (1.45 - 1.79)			<0.0001
Metastatic cancer	1.76 (1.43 - 2.18)			<0.0001
Weight loss	1.40 (1.23 - 1.59)			<0.0001
Electrolytes disorders	1.12 (1.02 - 1.23)			0.02
Liver disease	1.79 (1.39 - 2.30)			<0.0001
Alcoholism	0.59 (0.33 - 1.03)			0.06
Renal failure	1.48 (1.34 - 1.64)			<0.0001
Hypothyroidism	0.83 (0.71 - 0.97)			0.02
Psychiatric diseases	0.93 (0.71 - 1.24)			0.64
Depression	0.77 (0.65 - 0.91)			0.002
Median household income				
1st quartile	1.01 (0.88 - 1.15)			0.95
2nd quartile	0.97 (0.85 - 1.11)			0.66
3rd quartile	1.06 (0.94 - 1.21)			0.35
4th quartile	Referent			
Primary insurance				
Medicare/Medicaid	Referent			
Private including HMO	1.38 (1.20 - 1.58)			<0.0001
Uninsured/Self-pay	2.69 (2.18 - 3.30)			<0.0001
Hospital bed size				
Small	1.31 (1.12 - 1.54)			0.001
Medium	1.27 (1.12 - 1.44)			0.0003
Large	Referent			
Hospital type				
Rural	2.26 (1.88 - 2.72)			<0.0001
Urban/Non-teaching	1.38 (1.23 - 1.56)			<0.0001
Teaching	Referent			
Hospital region				
Northeast	Referent			
Midwest	0.81 (0.69 - 0.95)			0.01
South	1.02 (0.89 - 1.18)			0.74
West	0.85 (0.72 - 1.01)			0.06

Predictors of APRDRG disability

Several predictors like increasing age (OR: 1.14; 95% CI: 1.11-1.18, P <0.0001), Black race (OR: 1.12; 95% CI: 1.02-1.23, P <0.0001), and multiple comorbidities were associated with higher odds of disability. Moreover, rural hospitals and urban/non-teaching hospitals were associated with higher odds of disability than teaching hospitals. Table 3 describes the detailed statistics.

**Table 3:** Predictors of disability among multiple myeloma patients HMO: health maintenance organization

Independent variable/characteristic	Odds ratio (95% CI)	P-value
Age (10-year increase)	1.14 (1.11 - 1.18)	<0.0001
Gender		
Male	1.08 (1.03 - 1.14)	0.004
Female	Referent	
Race		
White	Referent	
Black	1.12 (1.02 - 1.23)	0.02
Hispanic	1.08 (0.96 - 1.21)	0.21
Others	0.97 (0.81 - 1.17)	0.75
Comorbidities		
Obesity	1.43 (1.28 - 1.60)	<0.0001
Hypertension	1.12 (1.06 - 1.19)	0.0001
Diabetes mellitus	0.86 (0.80 - 0.93	0.0001
Congestive heart failure	0.83 (0.81 - 0.84)	<0.0001
Hospital-onset pneumonia	7.00 (6.06 - 8.08)	<0.0001
Septicemia	1.72 (1.46 - 2.02)	<0.0001
History of chronic pulmonary disease	1.35 (1.24 - 1.47)	<0.0001
Pulmonary circulatory disease	6.15 (4.79 - 7.89)	<0.0001
Peripheral vascular disease	1.50 (1.24 - 1.81)	<0.0001
Neurologic disease	1.63 (1.44 - 1.85)	<0.0001
Paralysis	4.77 (3.88 - 5.86)	<0.0001
Coagulopathy	2.23 (1.99 - 2.50)	<0.0001
Metastatic cancer	4.07 (3.38 - 4.89)	<0.0001
Weight loss	3.56 (2.50 - 5.07)	<0.0001
Electrolytes disorders	2.80 (2.54 - 3.08)	<0.0001
Liver disease	1.97 (1.60 - 2.42)	<0.0001
Alcoholism	1.65 (1.29 - 2.11)	<0.0001
Renal failure	3.24 (3.01 - 3.49)	<0.0001
Hypothyroidism	1.10 (1.01 - 1.20)	0.03
Psychiatric diseases	1.35 (1.14 - 1.60)	0.0004
Depression	1.20 (1.10 - 1.31)	<0.0001
Median household income		
1st quartile	0.96 (0.88 - 1.05)	0.38
2nd quartile	0.94 (0.86 - 1.02)	0.15
3rd quartile	0.98 (0.90 - 1.06)	0.58
4th quartile	Referent	
Primary insurance		
Medicare/Medicaid	Referent	
Private including HMO	0.78 (0.72 - 0.84)	<0.0001
Uninsured/Self-pay	0.91 (0.80 - 1.05)	0.2
Hospital bed size		
Small	0.99 (0.81 - 1.20)	0.9
Medium	1.40 (1.23 - 1.60)	<0.0001
Large	Referent	
Hospital type		
Rural	1.37 (1.17 - 1.61)	<0.0001
Urban/Non-teaching	1.83 (1.66 - 2.03)	<0.0001
Teaching	Referent	
Hospital region		
Northeast	Referent	
Midwest	0.79 (0.65 - 0.96)	0.02
South	0.93 (0.81 - 1.07)	0.33
West	0.92 (0.78 - 1.09)	0.35

## Discussion

In this nationwide inpatient sample study, the burden of hospitalizations due to MM increased from 17,100 (8.7%) in 2007 to 19,490 (9.9%) in 2017. Vakiti et al. also found uptrending MM hospitalizations from 2002 to 2014 [3]. After the year 2014, we found a significant uptrend in the number of annual MM hospitalizations (Figure 1). The trend can likely be explained by the current understanding of the epidemiology of MM. Multiple myeloma is recognized as a disease of the elderly, with an average age of onset of 70-75 years [17]. With an overall increase in the aging population and the availability of newer treatment modalities like proteasome inhibitors, immunomodulatory drugs, allogenic bone marrow transplants, and targeted antibodies, overall survival rates among patients with MM have climbed in the US [18,19]. According to a study done in 2018, the five-year survival of patients in the 65-70 age group is 50%, which increases to 60% for younger patients [20]. Similar trends in survival rates also hold true for other countries like Sweden, Switzerland, and China [21-23].

Multiple myeloma is a disease with male preponderance; the incidence in males is approximately 1.5 times higher than that in females (7.8 per 100,000-person year for males compared to 5.1 per 100,000-person year for males) [20]. Our data analysis similarly shows higher hospitalization rates for males than females. Hypertension, electrolyte imbalance, renal failure, and coagulation abnormalities were the top co-morbidities associated with MM hospitalizations [24]. Most of the MM hospitalizations were in large, teaching hospitals, with the majority being emergent or urgent transfers from other health centers. This is likely because of the complications associated with this disease and the medications used for treatment, which require advanced treatment like plasmapheresis, dialysis, multiple blood transfusions, and the need for specialists [25]. In our study, MM hospitalizations were distributed among all quartiles of median household income, but coverage was predominantly provided by Medicare/Medicaid (58%), with self-pay being 6% (Table 1).

From 2007 to 2017, the mortality rate in MM hospitalizations dropped from 8.4% to 4.9%. Usui and colleagues identified that the annual percent change (APC) in mortality after the year 2002 in the US is -2.0% [26]. In recent times, the popularity of medications and their combinations (thalidomide, bortezomib, and lenalidomide antibodies like daratumumab and elotuzumab) with superior profiles and different toxicity profiles has had a significant contribution to lowering mortality and improving the overall survival rate [26,27]. Despite the development and availability of all these novel agents, surprisingly, the median length of stay was 12 days from 2007 to 2017. From 2007 to 2017, most patients were increasingly discharged home. Discharge-to-home increased from 71.19% to 77.3%, and the discharge-to-facility rate decreased from 20.39% to 17.35%. The results of increasing disposition to home can be presumed to be a better recovery.

We noted that the presence of hospital-onset pneumonia carries the highest risk of mortality, with an OR of 4.18. There are multiple reasons for increased susceptibility to infections among MM patients. Deranged antibody production, impaired renal function, suppressed immunity, and the use of immunosuppressants are significant [28,29]. Most of the treatments used in autologous MM stem cell transplants and novel anti-MM medications increase the risk of infection [30]. Use of daratumumab can cause natural killer cell depletion, causing increased infectious complications, and lenalidomide has a relative risk of 2.2, causing high-grade infection and ultimately increasing early mortality [31,32]. Infection can occasionally be the first presentation, and pneumonia is noted to have the highest incidence among all [33]. As noted in our results, the presence of metastatic cancer, liver disease, renal failure, congestive heart failure, neurological disease including paralysis, coagulopathy, weight loss, and electrolyte disorder are the significant co-morbidities associated with a higher risk of mortality. In addition to direct causes leading to death, these co-morbidities compromise patients' ability to tolerate treatment, indirectly causing poor survival. The predictors of in-hospital mortality represented by our study are in alignment with other studies, which have identified infections, renal disorders, and cardiac disorders as common MM-related causes of death [34,35].

In our study, obese MM patients did not have a statistically increased risk of mortality; this is in contradiction to a previous meta-analysis that noted that obesity is associated with a higher relative risk of death [36]. A large Portuguese study has shown the odds of in-hospital mortality are lower in depressed patients, but the length of hospital stay is longer [37]. Similarly, in real-world data, overall survival from chronic obstructive pulmonary disease (COPD) in multiple myeloma is worse, which contradicts our finding of a low odds ratio of mortality in hospitalized patients [38,39]. The reason for these findings remains unclear.

Although new therapeutic agents have lengthened survival in MM, their benefit in an older population is doubtful [40]. Older adults tend to have advanced disease, are ineligible for autologous stem cell transplants, and are likely to be intolerant of the toxicity of chemotherapy, causing high mortality [41]. These findings are in accordance with our results, which show worsening mortality with the rising age of MM patients. In our study, we found that there was a statistically insignificant increase in mortality for Black and Hispanic populations. Some of the single-center studies showed that Black patients have a poorer prognosis, while few failed to show those differences in the setting of equal access to the health system [42,43]. Increased mortality during hospitalization significantly depends on insurance coverage; we found that the odds of mortality were 2.6 higher for uninsured or self-paying MM patients in comparison to insured MM patients. Fiala et al. similarly found that MM patients with low socioeconomic status had a 54% increase in mortality rate relative to patients with high socioeconomic status [44]. We found that the odds of mortality were lower among large, teaching hospitals, which was likely secondary to the availability of resources to manage complicated patients with advanced MM.

We also investigated APR-DRG with disabilities. As discussed above, advanced age and Black race with multiple co-morbidities and hospitalization in rural, non-teaching hospitals are associated with higher disability (Table 3). Identifying risk factors associated with increased mortality and disability in a group of patients will help tailor patient-specific treatments. Some researchers have created a prediction matrix for early mortality that uses variables like age, performance status, renal failure, mobility score, staging, and thrombocytopenia [45]. Our study has extensively evaluated multiple variables, which can help other researchers develop a new prediction matrix to identify MM patients at higher risk of mortality and disability during hospitalizations.

Limitations exist in our study. Our study is a retrospective study. The selection of the population depends on the ICD-9/10-CM diagnoses codes for MM, which might have confounded our results. Our study provided an OR of inpatient mortality and disability, which is not enough to establish a causal relationship. Despite these limitations, our study has several strengths. This is the first nationwide study looking for trends and outcomes of MM hospitalizations from 2007 to 2017. A large sample size has yielded a high power to study results. Our study can establish a prediction matrix for mortality and disability using variables like age, multiple different co-morbidities, socioeconomic status, race, and type of hospital.

## Conclusions

Our study shows that MM hospitalizations are increasing annually. In-hospital mortality continues to decrease, but the length of stay has remained stable. Poorer outcomes regarding mortality and APR-DRG disability depend on increasing age, infection, renal failure, and other co-morbidities. Mortality and disability also depend on socioeconomic status, insurance status, and the type of hospital. Further prospective studies are warranted for better risk stratification and improvement in MM outcomes by building a new prediction matrix using the variables that we have identified.
